# Structural characterization and DFT study of bis­{(*S*)-2-[(2-hy­droxy­benz­yl)amino]-3-(4-hy­droxy­phen­yl)propano­ato-κ^2^ *N*,*O*}(1,10-phenanthroline-κ^2^ *N*,*N*′)cadmium(II) tetra­hydrate

**DOI:** 10.1107/S205698901801157X

**Published:** 2018-08-24

**Authors:** Md. Serajul Haque Faizi, Necmi Dege, James Pogrebetsky, Turganbay S. Iskenderov

**Affiliations:** a Department of Chemistry, Langat Singh College, Babasaheb Bhimrao Ambedkar Bihar University, Muzaffarpur, Bihar, India; b Ondokuz Mayis University, Arts and Sciences Faculty, Department of Physics, 55139 Samsun, Turkey; c Department of Chemistry, Taras Shevchenko National University of Kyiv, 64 Volodymyrska Str., 01601 Kiev, Ukraine

**Keywords:** crystal structure, Cd^II^ complex, distorted octa­hedral geometry, O—H⋯O hydrogen bonding, π–π stacking inter­actions

## Abstract

In the title compound, [Cd(C_16_H_16_O_3_)_2_(C_12_H_8_N_2_)]·4H_2_O, the Cd ion lies on a twofold rotation axis and is chelated by two monodeprotonated residues of the l-tyrosine-derived ligand (*S*)-2-[(2-hy­droxy­benz­yl)amino]-3-(4-hy­droxy­phen­yl)propanoic acid (**L**) in a κ^2^ *N*,*O* amino acid chelating mode, exhibiting an (*N*,*N*′)-*trans* disposition, and by 1,10-phenanthroline in a κ^2^ *N*,*N*′ mode. The latter ligand is also located about the twofold rotation axis that bisects the central six-members ring. The phenolic groups remain protonated and non-coordinating and take part as acceptors in the intra­molecular hydrogen bonds with the amino groups of the acido ligands. The Cd ion is six-coordinated in a distorted octa­hedral environment. In the crystal, O—H⋯O hydrogen bonds result in the formation of three-dimensional network structures. The title complex has also been characterized by IR and ^1^H NMR spectroscopy and DFT studies. The crystal studied was refined as an inversion twin.

## Abstract 2

In the crystal, the Cd^II^ atom, located on a twofold rotation axis, is coordinated by three chelating ligands, leading to a distorted octa­hedral CdN_4_O_2_ coordination sphere.

## Chemical context   

Schiff bases are widely known as an important class of organic compounds and ligands in coordination chemistry. In recent years they have found applications in the fields of analytical chemistry, medicine and biological processes, displaying anti­fungal, anti­bacterial and anti­cancer activities (Przybylski *et al.*, 2009 ▸; Dhar & Taploo, 1982 ▸). Such systems are considered important ligands for coordination and supra­molecular compounds (Moroz *et al.*, 2012 ▸). Coordination complexes with Schiff bases have attracted the inter­est of researchers in the areas of pharmaceutical, agriculture and industrial chemistry (Anis *et al.*, 2013 ▸). However, the use of Schiff base ligand systems having additional polar donor functions on contrary oximes (Sliva *et al.*, 1997 ▸; Penkova *et al.*, 2010 ▸; Pavlishchuk *et al.*, 2010 ▸) is limited because of their enhanced reactivity or instability under complex formation (Casella & Gullotti, 1983 ▸). For example, Schiff bases derived from amino­hydroxamic acids undergo spontaneous cyclization resulting in the formation of 2-substituted 3-hy­droxy­imidazolidine-4-ones (Iskenderov *et al.*, 2009 ▸). One of the ways to overcome this drawback is the reduction of such compounds to amines. The formed ligands are more conformationally flexible at the coordination site, thus not necessarily forming planar chelate rings (Koh *et al.*, 1996 ▸). In recent years it has also been found that phenanthroline, another ligand used in this study, has extensive important roles in a variety of fields (Faizi & Sharkina, 2015 ▸; Faizi *et al.*, 2017 ▸). In this paper we report the synthesis and structure of a new cadmium complex with an l-tyrosine-derived ligand synthesized by the reduction of a Schiff base precursor.
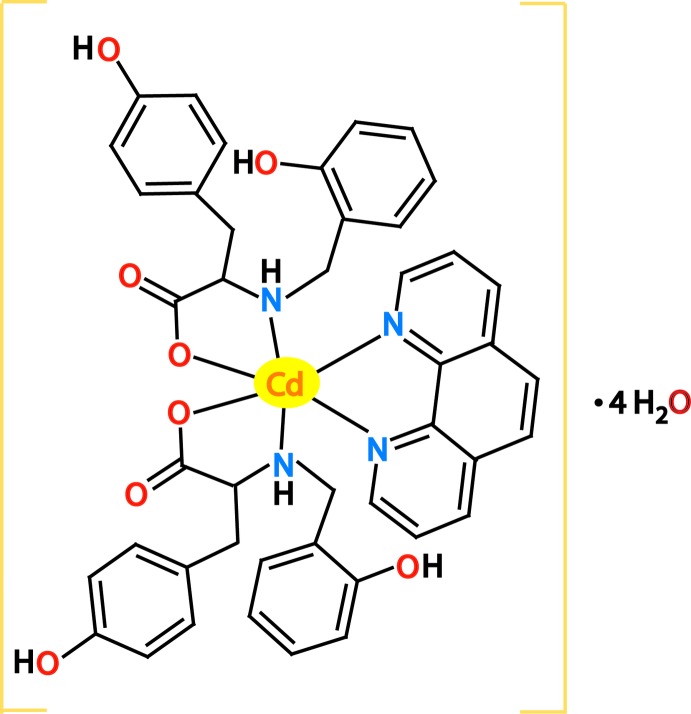


## Structural commentary   

**Figure 1:**
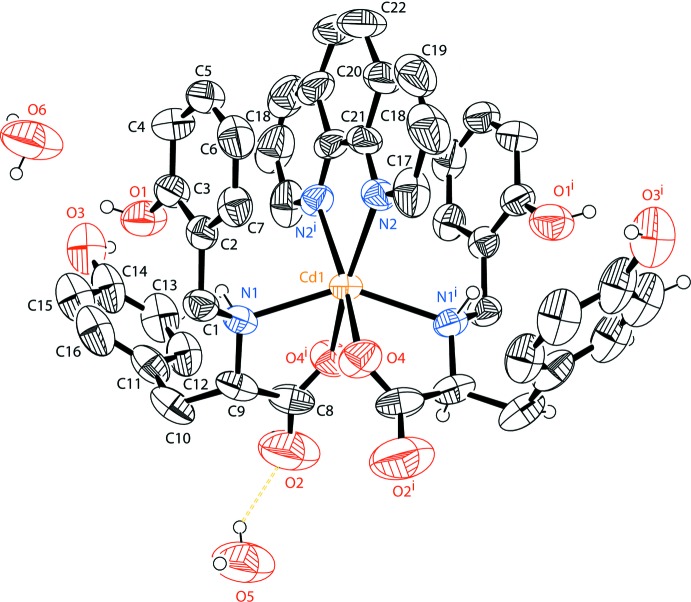
The mol­ecular structure of the title compound, showing the atom labelling for the asymmetric unit. Displacement ellipsoids are drawn at the 50% probability level. The unabelled atoms are related to the labelled atoms by symmetry operation *y*, *x*, −*z*

The asymmetric unit of the title compound contains one half of the mononuclear complex of the mononuclear complex [Cd(**L**-H)_2_(phen)] and two water mol­ecules of solvation (Fig. 1 ▸). The central Cd^II^ atom is located on a twofold rotation axis and coordinated by three chelating ligands, leading to a distorted octa­hedral CdN_4_O_2_ coordination sphere. The mixed-ligand complex contains one neutral phenanthroline ligand, bisected by the twofold rotation axis, and two residues of monodeprotonated tyrosine-derived ligands. The latter are coordinated in a κ^2^ *N*,*O* classical amino acid chelating mode and have a C9 chiral atom, exhibiting an (*N*,*N*′)-*trans* disposition. The Cd—O and Cd—N bond lengths are similar, being 2.325 (5), 2.335 (6) and 2.323 (6) Å for Cd1—O4, Cd1—N1 and Cd1—N2, respectively. All three ligands form five-membered chelate rings. Unlike the chelate ring formed by the phenanthroline ligand which is virtually planar, the one created by the **L** residue exhibits an envelope conformation with the deviation of the Cd atom from the mean plane defined by the other four atoms being 1.0692 (3) Å. The N—Cd—O and N—Cd—N bite angles are 70.5 (2) and 72.0 (4)°, respectively. The phenolic O—H group remains protonated and non-coordinating, albeit participating in an extensive inter­molecular hydrogen-bonding network. An intra­molecular N1—H1⋯O1 hydrogen bond (Table 1 ▸) occurs between the amino and phenolic groups of the same acido ligand.

## Supra­molecular features   

**Figure 2:**
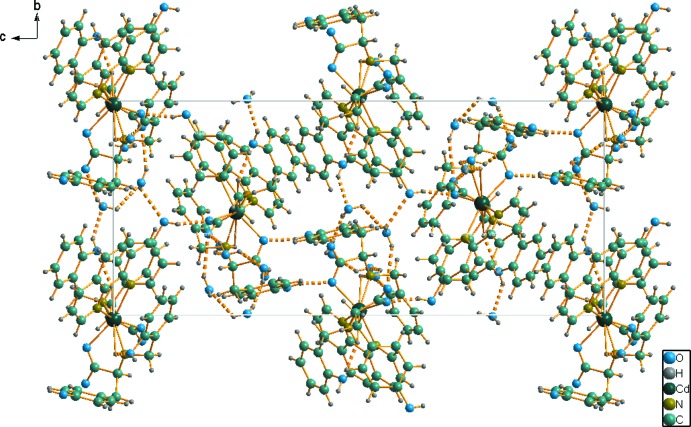
The crystal packing of the title compound viewed along the *a* axis. Hydrogen bonds are shown as dashed lines (see Table 1 ▸ for details).

The crystal structure of title compound is stabilized by inter­molecular O—H⋯O hydrogen bonds in which the phenolic groups of the **L** ligand and the water mol­ecules act as both donors and acceptors (Table 1 ▸, Fig. 2 ▸). Hydrogen bonds formed by the water mol­ecules link the neighboring complex mol­ecules, forming a three-dimensional structure. π–π inter­actions take place between the central ring of phenanthroline and the C2–C7 aromatic rings of two tyrosine-derived ligands with centroid-to-centroid separations of 3.938 (6) Å.

## DFT study   

**Figure 3:**
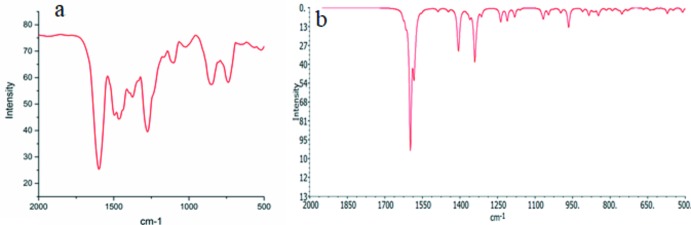
(a) Experimental vibrational spectrum and (*b*) B3LYP/DFT simulated vibrational spectrum.

Density functional theory (DFT) calculations were performed to investigate the electronic structure and characteristic vibrations. The calculated frequencies were found within the range, shown in Table 2 ▸. Two factors could be responsible for the shift between the experimental and computed spectra (Fig. 3 ▸). The first is the environmental factor as the DFT calculations were performed for the gas phase while the experimental data were obtained for the solid state. The second reason for the shift is that the calculated values are only harmonic frequencies while the experimental values contain both harmonic and anharmonic vibrational frequencies, but the pattern of the spectra appear to be quite similar in both cases, which validates the experimental vibrational spectrum. Some animated images of the characteristic vibrations with displacement vector are given in supporting information.

## Frontier mol­ecular orbital analysis   

**Figure 4:**
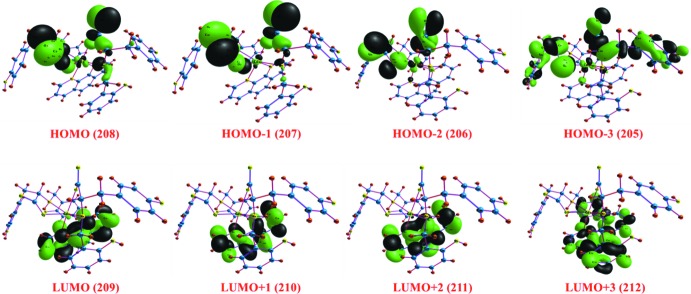
HOMO and LUMO frontier molecular orbitals with respective mol­ecular orbital number.

The LUMO and HOMO orbital energy parameters are accountable to a significant extent for the charge transfer, chemical reactivity and kinetic/thermodynamic stability of a mol­ecule. Metal complexes with a small energy gap (Δ*E*) between the HOMO and LUMO values are more polarizable, thereby acting as soft mol­ecules with a higher chemical reactivity. However, complexes with large energy gap offer greater stability and lower chemical reactivity than those with a small HOMO–LUMO energy gap. The DFT study revealed that the HOMO, HOMO-1, HOMO-2 and HOMO-3 energies are localized on the N1, N4, O2, O3, O6, O7, C8, C9, C35, C36 and C37 atoms of the amino acid ligand, partially localized on the Cd centre, namely *dx* ^2^ − *y* ^2^, as shown in Fig. 4 ▸. In contrast, LUMO, LUMO+1, LUMO+2 and LUMO+3 are totally delocalized over phenanthroline moiety. It could be said that the HOMO and LUMO are mainly composed of σ and π-type orbitals, respectively, and that intra­molecular charge transfer occurred from the amino acid moiety to the phenanthroline moiety. The LUMO–HOMO gap of the complex was calculated to be 2.30 eV. The frontier mol­ecular orbital energies are given in Table 3 ▸.

## Hirshfeld surface analysis   

**Figure 5:**
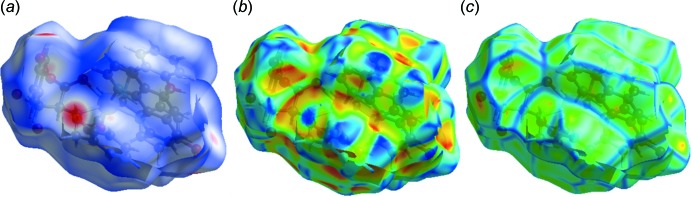
Hirshfeld surfaces mapped over (*a*) *d*_norm_, (*b*) shape index and (*c*) curvedness.

**Figure 6:**
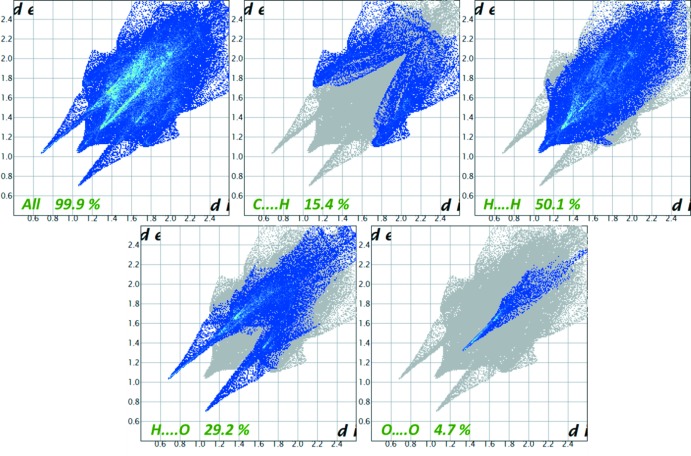
The two-dimensional fingerprint plots of inter­atomic inter­actions showing the percentage contributions to the Hirshfeld surface.

The Hirshfeld surfaces of the title compound are illustrated in Fig. 5 ▸, depicting surfaces that have been mapped over a *d*_norm_ range of −0.5 to 1.5 a.u., shape index (−1.0 to 1.0 a.u.) and curvedness (−4.0 to 0.4 a.u.). The *d*_norm_ surface has a red–white–blue colour scheme, whereas deep-red spots highlight shorter contacts *i.e.* hydrogen bonding. The white areas represent contacts around the van der Waals separation, such as H⋯H contacts, and the blue regions are devoid of such close contacts. On the Hirshfeld surface mapped with the shape-index function, one can examine both red regions corresponding to C—H⋯π inter­actions as well as ‘bow-tie patterns’, which indicate the presence of aromatic stacking (π–π) inter­actions. The curvedness surface indicates that the electron density of the surface curves around the mol­ecular inter­actions. The fingerprint plots, presented in Fig. 6 ▸, can be decomposed to highlight particular atom-pair close contacts. This itemization allows visualization of the contributions from different inter­action types, which overlap in the full fingerprint. For the title compound, the proportions of H⋯H, C⋯H, H⋯O and O⋯O inter­actions comprise 50.1%, 15.4%, 29.2% and 4.7%, respectively, of the total Hirshfeld surface for each mol­ecule.

## Database survey   

A search in the Cambridge Structural Database (Version 5.39, last update February 2018; Groom *et al.*, 2016 ▸) for structures with a Cd^II^ ion coordinated by 2-hy­droxy­benzyl derivatives of amino acids yielded only one hit (refcode WARLIL; Lou *et al.*, 2005 ▸), a mononuclear complex with *N*-(2-hy­droxy­benz­yl)-d,l-aspartic acid. In this complex, the doubly deprotonated (by the phenolic and β-carb­oxy­lic groups) residue of the ligand is coordinated in an (*O*,*N*,*O*′)-tridentate mode including the phenolic oxygen, unlike the title compound in which the phenolic group is non-coordinating. The second oxygen atom of the β-carb­oxy­lic group bridges the neighboring mononuclear Cd units into a one-dimensional chain. In addition, there are few structures of complexes with zinc or cadmium analogues (refcodes AZIROQ, AZIRUW, NOLYIW, NOLYOC) with 2-hy­droxy­benzyl derivatives of alanine. In all these complexes, the ligand is also coordinated in an (*O*,*N*,*O*′)-tridentate manner, with an additional μ_2_-function of the phenolic oxygen, which results in the formation of a Zn_2_O_2_ binuclear core in all cases (Lou *et al.*, 2004 ▸; Ranford *et al.*, 1998 ▸).

## Synthesis and crystallization   

**Synthesis of (** ***S*** **)-2-[(2-hy­droxy­benz­yl)amino]-3-(4-hy­droxy­phen­yl)propanoic acid (L)**

A methano­lic solution of *o*-salicyl­aldehyde (1.18 g, 5.51 mmol) was added dropwise to a stirring solution of l-tyrosine (1.00 g, 5.52 mmol) and LiOH·H_2_O (0.23 g, 5.50 mmol) in methanol (25 mL). Stirring was continued for 2 h, followed by the addition of sodium borohydride (0.21 g, 5.55 mmol) with further stirring for 1 h. The solvent was evaporated and the resulting sticky mass was dissolved in water and acidified with dilute HCl. The pH of the solution was maintained between 5–7. The ligand precipitated as a brown solid. It was washed thoroughly with water and MeOH after filtration and dried in a vacuum desiccator. Yield 1.60 g (76%). *m*/*z* (ESI–MS, [*M* − H]^−^) 379.087 (calculated 379.084). IR (KBr, cm^−1^), ν(COO)_asym_ 1579 (*s*), ν(COO)_sym_ 1394 (*s*). ^1^H NMR (CD_3_OD, ppm): H_cp.o_ (4.10, *d*, 1H), H_cp.o_ (4.13, *d*, 1H), H_cp_ (3.99, *s*, 5H), H_cp.m_ (4.03, *s*, 2H), H_a_ (3.51, *d*, 1H, *J*_a,a_ = 12.8 Hz), H_a,c′_ (3.22, *d*, 1H), H_b_ (3.31, *m*, 1H), H_c_ (2.94, *dd*, 1H), H_c,c′_ (2.67, *dd*, 1H), H_d,d′_ (6.96, *d*, 2H), H_e,e′_ (6.59, *d*, 2H). As a result of geminal coupling, H_c_ split into two non-equivalent H_c_ and H_c′_.

**Synthesis of [Cd(L-H)_2_(phen)]·4H_2_O**

A methano­lic solution of Cd(NO_3_)_2_·6H_2_O (0.107 g, 0.348 mmol) was added to a stirred 15 ml methano­lic solution of **L** (0.200 g, 0.696 mmol) and NaOH (0.028 g, 0.696 mmol), followed by addition of phenanthroline monohydrate (0.063 g, 0.348 mmol) in 5 ml of MeOH. A clear solution was obtained. After 20 minutes stirring a precipitate appeared. The reaction mixture was evaporated under reduced pressure. The residue was washed with water and subsequently diethyl ether, and finally dried under vacuum. Prismatic crystals suitable for X-ray data collection were obtained by slow evaporation of methanol. Empirical formula [Cd(**L**)_2_(phen)]·4H_2_O (**2**). Yield 49%. [Cd(**L**)_2_(phen)]·4H_2_O: IR (KBr, cm^−1^) ν(COO)_assym_ 1651, ν(COO)_sym_ 1381, ν(phenolic, CO) 1250. ^1^H NMR [Cd(**L**)_2_(phen)]·4H_2_O] (DMSO, 400 MHz. ppm): 2.4 (*s*, *br*, 1H_g_), 2.5 (*s*, *br*, 1H_g_^’^), 2.8 (*s*, *br*, 1H_f_), 3.1 (*s*, *br*, 1H_e_), 3.4 (*s*, *br*, 1H_e′_), 6.1 (*s*, *br*, 2H_a,c_), 6.5 (*s*, *br*, 2H_b,d_), 7.0 (*s*, *br*, 4H), 7.9 (*s*, *br*, 2H_n_), 8.0 (*s*, *br*, 2H_m_), 8.7 (*s*, *br*, 2H_l_), 9.0 (*s*, *br*, 2H_k_). ESI–Mass (-ve) 929.2 (calculated 929.2).

## Refinement   

Crystal data, data collection and structure refinement details are summarized in Table 4 ▸. The O—H H atoms were located in a difference-Fourier map and constrained to ride on their parent atoms, with O—H = 0.82 Å and with *U*_iso_(H) = 1.5*U*_eq_(O). All C-bound H atoms were positioned geometrically and refined using a riding model with C—H = 0.93 Å and with *U*_iso_(H) = 1.2*U*_eq_(C). The crystal studied was refined as an inversion twin.

## Supplementary Material

Crystal structure: contains datablock(s) I, global. DOI: 10.1107/S205698901801157X/xu5935sup1.cif

Structure factors: contains datablock(s) I. DOI: 10.1107/S205698901801157X/xu5935Isup2.hkl

1487 +COO symmetrical stretch. DOI: 10.1107/S205698901801157X/xu5935sup3.gif

1237 +C-N stretch. DOI: 10.1107/S205698901801157X/xu5935sup4.gif

885 +NH wagging. DOI: 10.1107/S205698901801157X/xu5935sup5.gif

756 +CH rocking. DOI: 10.1107/S205698901801157X/xu5935sup6.gif

1598 +COO antisymmetrical stretch. DOI: 10.1107/S205698901801157X/xu5935sup7.gif

3414 +NH stretching. DOI: 10.1107/S205698901801157X/xu5935sup8.gif

CCDC reference: 1534110

Additional supporting information:  crystallographic information; 3D view; checkCIF report

## Figures and Tables

**Table 1:** Hydrogen-bond geometry (Å, °)

*D*—H⋯*A*	*D*—H	H⋯*A*	*D*⋯*A*	*D*—H⋯*A*
N1—H1⋯O1	1.02	2.27	2.992 (10)	126
O6—H6*A*⋯O2^i^	0.83	2.51	3.276 (18)	152
O6—H6*B*⋯O3	0.83	2.43	2.851 (16)	112
O5—H5*B*⋯O2	0.82	2.00	2.675 (15)	140
O5—H5*A*⋯O6^ii^	0.83	2.31	2.709 (17)	110
O1—H1*C*⋯O5^iii^	0.82	1.98	2.652 (11)	138
O3—H3⋯O4^iv^	0.82	1.86	2.640 (9)	159

Symmetry codes: (i) 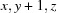; (ii) 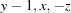; (iii) 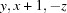; (iv) 
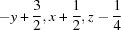
.

**Table 2:** Some selected experimental and calculated wavenumbers (cm^−1^)

Vibrational band	Experimental	Calculated
ν(N—H) stretching	3402	3414
ν(COO) anti-symmetrical stretching	1597	1598
ν(COO) symmetrical stretching	1459,1376	1487, 1396
ν(C—N) stretching	1266	1237
ν(N—H) wagging	853	885
ν(C—H) rocking	722	756

**Table 3:** Calculated frontiermolecular orbital energies (eV)

FMO	Energy
LUMO+3	−0.90
LUMO+2	−1.68
LUMO+1	−2.73
LUMO	−2.78
HOMO	−5.08
HOMO-1	−5.15
HOMO-2	−5.82
HOMO-3	−5.89
LUMO–HOMO	2.30

**Table 4:** Experimental details

Crystal data	
Chemical formula	[Cd(C_16_H_16_O_3_)_2_(C_12_H_8_N_2_)]·4H_2_O
*M*_r_	937.26
Crystal system, space group	Tetragonal, *P*4_3_2_1_2
Temperature (K)	296
*a*, *c* (Å)	12.4171 (2), 28.4151 (10)
*V* (Å^3^)	4381.2 (2)
*Z*	4
Radiation type	Mo *K*α
μ (mm^−1^)	0.57
Crystal size (mm)	0.20 × 0.14 × 0.11

Data collection	
Diffractometer	Bruker SMART CCD area detector
No. of measured, independent and observed [*I* > 2σ(*I*)] reflections	28145, 4310, 3599
*R*_int_	0.049
(sin θ/λ)_max_ (Å^−1^)	0.617

Refinement	
*R*[*F* ^2^ > 2σ(*F* ^2^)], *wR*(*F* ^2^), *S*	0.053, 0.159, 1.04
No. of reflections	4310
No. of parameters	260
No. of restraints	18
H-atom treatment	H-atom parameters constrained
Δρ_max_, Δρ_min_ (e Å^−3^)	0.75, −0.73
Absolute structure	Refined as an inversion twin
Absolute structure parameter	0.02 (7)

Computer programs: *SMART* (Bruker, 2012 ▸), *SAINT* (Bruker, 2012 ▸), *SHELXTL* (Sheldrick, 2008 ▸), *SHELXL2016* (Sheldrick, 2015 ▸).

## supplementary crystallographic information

### Crystal data

**Table:**

[Cd(C_16_H_16_O_3_)_2_(C_12_H_8_N_2_)]·4H_2_O	*D*_x_ = 1.421 Mg m^−^^3^*D*_m_ = 1.421 Mg m^−^^3^*D*_m_ measured by ?
*M**_r_* = 937.26	Mo *K*α radiation, λ = 0.71073 Å
Tetragonal, *P*4_3_2_1_2	Cell parameters from 5678 reflections
*a* = 12.4171 (2) Å	θ = 2.7–21.8°
*c* = 28.4151 (10) Å	µ = 0.56 mm^−^^1^
*V* = 4381.2 (2) Å^3^	*T* = 296 K
*Z* = 4	Prism, colorless
*F*(000) = 1936	0.20 × 0.14 × 0.11 mm

### Data collection

**Table:**

Bruker SMART CCD area detector diffractometer	3599 reflections with *I* > 2σ(*I*)
Radiation source: sealed tube	*R*_int_ = 0.049
Graphite monochromator	θ_max_ = 26.0°, θ_min_ = 2.2°
phi and ω scans	*h* = −15→15
28145 measured reflections	*k* = −14→15
4310 independent reflections	*l* = −33→35

### Refinement

**Table:**

Refinement on *F*^2^	Hydrogen site location: mixed
Least-squares matrix: full	H-atom parameters constrained
*R*[*F*^2^ > 2σ(*F*^2^)] = 0.053	*w* = 1/[σ^2^(*F*_o_^2^) + (0.1136*P*)^2^ + 0.4952*P*] where *P* = (*F*_o_^2^ + 2*F*_c_^2^)/3
*wR*(*F*^2^) = 0.159	(Δ/σ)_max_ = 0.001
*S* = 1.03	Δρ_max_ = 0.75 e Å^−^^3^
4310 reflections	Δρ_min_ = −0.73 e Å^−^^3^
260 parameters	Absolute structure: Refined as an inversion twin
18 restraints	Absolute structure parameter: 0.02 (7)

### Special details

**Table:**

Geometry. All esds (except the esd in the dihedral angle between two l.s. planes) are estimated using the full covariance matrix. The cell esds are taken into account individually in the estimation of esds in distances, angles and torsion angles; correlations between esds in cell parameters are only used when they are defined by crystal symmetry. An approximate (isotropic) treatment of cell esds is used for estimating esds involving l.s. planes.
Refinement. Refined as a 2-component inversion twin.

### Fractional atomic coordinates and isotropic or equivalent isotropic displacement parameters (Å^2^)

**Table:**

	*x*	*y*	*z*	*U*_iso_*/*U*_eq_	
O5	0.5065 (7)	0.7805 (10)	−0.0219 (6)	0.192	
H5B	0.536098	0.833414	−0.033121	0.288*	
H5A	0.492616	0.795873	0.005957	0.288*	
O6	0.7175 (8)	1.6168 (10)	−0.0556 (5)	0.192	
H6A	0.710747	1.678849	−0.066486	0.288*	
H6B	0.655019	1.593062	−0.053790	0.288*	
Cd1	0.97964 (3)	0.97964 (3)	0.000000	0.0540 (2)	
N1	0.8171 (5)	1.0589 (5)	0.0225 (2)	0.0623 (15)	
H1	0.817840	1.129105	0.003614	0.093*	
C9	0.7269 (6)	0.9904 (7)	0.0079 (3)	0.079 (3)	
H9	0.718656	0.935076	0.032265	0.095*	
C14	0.6231 (8)	1.3351 (11)	−0.0780 (3)	0.101 (4)	
C11	0.6180 (7)	1.1475 (10)	−0.0258 (3)	0.089 (3)	
C12	0.6472 (8)	1.1455 (10)	−0.0743 (3)	0.094 (3)	
H12	0.667411	1.081506	−0.088820	0.113*	
C15	0.5947 (9)	1.3356 (12)	−0.0318 (4)	0.107 (4)	
H15	0.572538	1.399858	−0.018019	0.128*	
C16	0.5977 (8)	1.2496 (13)	−0.0067 (4)	0.106 (4)	
H16	0.585881	1.255378	0.025537	0.127*	
C10	0.6175 (6)	1.0501 (10)	0.0045 (3)	0.095 (3)	
H10A	0.563903	1.000308	−0.007451	0.114*	
H10B	0.595478	1.070992	0.036001	0.114*	
C13	0.6449 (10)	1.2404 (12)	−0.0991 (4)	0.107 (4)	
H13	0.658674	1.239261	−0.131233	0.128*	
C3	0.9033 (7)	1.2746 (7)	0.0637 (3)	0.071 (2)	
C2	0.9025 (6)	1.1740 (7)	0.0832 (2)	0.0603 (18)	
C4	0.9866 (8)	1.3478 (7)	0.0716 (3)	0.083 (2)	
H4	0.984052	1.415892	0.058059	0.099*	
C7	0.9869 (7)	1.1450 (7)	0.1124 (3)	0.073 (2)	
H7	0.988546	1.077657	0.126667	0.088*	
C1	0.8112 (7)	1.0959 (7)	0.0718 (3)	0.067 (2)	
H1A	0.815395	1.034217	0.092652	0.080*	
H1B	0.742570	1.131201	0.077095	0.080*	
C6	1.0698 (8)	1.2195 (10)	0.1198 (3)	0.089 (3)	
H6	1.126603	1.200829	0.139472	0.107*	
C5	1.0699 (8)	1.3199 (9)	0.0987 (3)	0.083 (2)	
H5	1.126985	1.367210	0.103385	0.100*	
C17	1.1960 (8)	0.9751 (11)	0.0666 (4)	0.099 (3)	
H17	1.169971	0.909387	0.077427	0.119*	
C21	1.1830 (6)	1.1235 (7)	0.0185 (3)	0.076 (2)	
C20	1.2837 (9)	1.1652 (11)	0.0351 (5)	0.110 (4)	
C19	1.3356 (10)	1.1048 (14)	0.0710 (5)	0.114 (5)	
H19	1.399452	1.129307	0.084379	0.137*	
C18	1.2943 (10)	1.0187 (16)	0.0844 (5)	0.120 (5)	
H18	1.330129	0.979963	0.107608	0.144*	
O1	0.8199 (7)	1.2984 (6)	0.0340 (3)	0.102 (2)	
H1C	0.790761	1.354342	0.042646	0.152*	
O3	0.6285 (7)	1.4344 (8)	−0.1026 (2)	0.123 (3)	
H3	0.646900	1.423349	−0.129854	0.184*	
N2	1.1419 (5)	1.0314 (6)	0.0338 (2)	0.0660 (16)	
C8	0.7555 (8)	0.9297 (9)	−0.0391 (4)	0.093 (3)	
O2	0.6804 (10)	0.8780 (10)	−0.0575 (5)	0.192	
O4	0.9391 (6)	0.8474 (5)	0.0552 (2)	0.0880 (19)	
C22	1.3163 (12)	1.2687 (12)	0.0170 (5)	0.142 (8)	
H22	1.375144	1.302587	0.030868	0.171*	

### Atomic displacement parameters (Å^2^)

**Table:**

	*U*^11^	*U*^22^	*U*^33^	*U*^12^	*U*^13^	*U*^23^
O5	0.111	0.160	0.306	−0.036	−0.056	0.082
O6	0.111	0.160	0.306	−0.036	−0.056	0.082
Cd1	0.0535 (3)	0.0535 (3)	0.0550 (4)	−0.0104 (3)	−0.0039 (2)	0.0039 (2)
N1	0.049 (3)	0.067 (4)	0.071 (4)	−0.010 (3)	0.001 (3)	0.008 (3)
C9	0.055 (4)	0.097 (6)	0.084 (6)	−0.024 (4)	−0.008 (4)	0.017 (5)
C14	0.081 (6)	0.158 (11)	0.064 (5)	0.045 (7)	−0.015 (4)	−0.006 (6)
C11	0.051 (4)	0.140 (10)	0.075 (6)	−0.001 (5)	−0.006 (4)	0.000 (6)
C12	0.087 (6)	0.121 (9)	0.074 (6)	0.025 (6)	−0.012 (5)	−0.002 (6)
C15	0.089 (7)	0.166 (11)	0.065 (6)	0.030 (7)	−0.011 (5)	0.001 (6)
C16	0.072 (5)	0.184 (12)	0.061 (6)	0.030 (6)	−0.018 (5)	−0.016 (7)
C10	0.050 (4)	0.151 (9)	0.082 (6)	−0.015 (5)	−0.003 (4)	0.014 (7)
C13	0.109 (8)	0.151 (11)	0.060 (5)	0.033 (8)	−0.016 (5)	−0.004 (6)
C3	0.060 (4)	0.075 (5)	0.078 (5)	−0.005 (4)	0.000 (4)	0.002 (4)
C2	0.062 (4)	0.071 (4)	0.048 (4)	−0.001 (3)	0.008 (3)	−0.010 (3)
C4	0.088 (6)	0.075 (5)	0.086 (6)	−0.023 (5)	−0.006 (5)	0.004 (4)
C7	0.077 (5)	0.086 (5)	0.056 (4)	0.008 (5)	0.002 (4)	−0.010 (3)
C1	0.062 (4)	0.067 (4)	0.072 (5)	−0.008 (4)	0.007 (4)	0.009 (4)
C6	0.069 (5)	0.121 (9)	0.076 (6)	0.014 (6)	−0.019 (4)	−0.033 (6)
C5	0.069 (5)	0.084 (6)	0.097 (7)	−0.016 (5)	0.006 (5)	−0.015 (5)
C17	0.093 (7)	0.122 (8)	0.082 (6)	0.033 (7)	−0.025 (5)	−0.023 (6)
C21	0.060 (4)	0.088 (6)	0.079 (5)	−0.024 (4)	0.020 (3)	−0.037 (4)
C20	0.070 (6)	0.121 (9)	0.138 (10)	−0.032 (6)	0.033 (6)	−0.073 (8)
C19	0.071 (7)	0.153 (13)	0.119 (10)	−0.003 (8)	−0.005 (7)	−0.053 (10)
C18	0.079 (7)	0.173 (14)	0.107 (9)	0.021 (9)	−0.030 (6)	−0.038 (10)
O1	0.092 (5)	0.086 (5)	0.126 (6)	−0.014 (4)	−0.027 (5)	0.016 (4)
O3	0.131 (7)	0.151 (8)	0.086 (4)	0.041 (6)	−0.013 (4)	−0.004 (5)
N2	0.054 (3)	0.080 (4)	0.064 (3)	−0.008 (3)	−0.006 (3)	−0.019 (3)
C8	0.067 (5)	0.113 (8)	0.098 (7)	−0.036 (5)	−0.029 (5)	−0.007 (6)
O2	0.111	0.160	0.306	−0.036	−0.056	0.082
O4	0.119 (5)	0.070 (4)	0.075 (4)	−0.013 (3)	0.022 (4)	0.018 (3)
C22	0.100 (11)	0.134 (15)	0.19 (2)	−0.067 (12)	0.036 (10)	−0.068 (12)

### Geometric parameters (Å, º)

**Table:**

O5—H5B	0.8179	C3—O1	1.367 (11)
O5—H5A	0.8319	C3—C4	1.394 (12)
O6—H6A	0.8336	C2—C7	1.384 (12)
O6—H6B	0.8313	C2—C1	1.527 (10)
Cd1—N2	2.323 (6)	C4—C5	1.336 (14)
Cd1—N2^i^	2.323 (6)	C4—H4	0.9300
Cd1—O4^i^	2.325 (5)	C7—C6	1.399 (13)
Cd1—O4	2.325 (5)	C7—H7	0.9300
Cd1—N1	2.335 (6)	C1—H1A	0.9700
Cd1—N1^i^	2.335 (6)	C1—H1B	0.9700
N1—C9	1.466 (9)	C6—C5	1.383 (15)
N1—C1	1.475 (10)	C6—H6	0.9300
N1—H1	1.0237	C5—H5	0.9300
C9—C10	1.551 (13)	C17—N2	1.345 (12)
C9—C8	1.573 (13)	C17—C18	1.428 (17)
C9—H9	0.9800	C17—H17	0.9300
C14—C13	1.347 (17)	C21—N2	1.325 (11)
C14—C15	1.359 (14)	C21—C20	1.433 (13)
C14—O3	1.419 (14)	C21—C21^i^	1.483 (19)
C11—C16	1.402 (17)	C20—C19	1.42 (2)
C11—C12	1.426 (14)	C20—C22	1.442 (19)
C11—C10	1.485 (14)	C19—C18	1.25 (2)
C12—C13	1.372 (17)	C19—H19	0.9300
C12—H12	0.9300	C18—H18	0.9300
C15—C16	1.285 (17)	O1—H1C	0.8200
C15—H15	0.9300	O3—H3	0.8200
C16—H16	0.9300	C8—O4^i^	1.235 (12)
C10—H10A	0.9700	C8—O2	1.248 (13)
C10—H10B	0.9700	C22—C22^i^	1.28 (3)
C13—H13	0.9300	C22—H22	0.9300
C3—C2	1.367 (12)		
			
H5B—O5—H5A	106.3	C12—C13—H13	119.2
H6A—O6—H6B	105.0	C2—C3—O1	116.2 (8)
N2—Cd1—N2^i^	72.0 (4)	C2—C3—C4	122.4 (8)
N2—Cd1—O4^i^	162.0 (2)	O1—C3—C4	121.4 (8)
N2^i^—Cd1—O4^i^	96.0 (3)	C3—C2—C7	118.3 (8)
N2—Cd1—O4	96.0 (3)	C3—C2—C1	120.0 (8)
N2^i^—Cd1—O4	162.0 (2)	C7—C2—C1	121.6 (8)
O4^i^—Cd1—O4	98.5 (4)	C5—C4—C3	119.9 (9)
N2—Cd1—N1	121.3 (2)	C5—C4—H4	120.0
N2^i^—Cd1—N1	89.2 (2)	C3—C4—H4	120.0
O4^i^—Cd1—N1	70.5 (2)	C2—C7—C6	118.4 (9)
O4—Cd1—N1	85.7 (2)	C2—C7—H7	120.8
N2—Cd1—N1^i^	89.2 (2)	C6—C7—H7	120.8
N2^i^—Cd1—N1^i^	121.3 (2)	N1—C1—C2	111.3 (6)
O4^i^—Cd1—N1^i^	85.7 (2)	N1—C1—H1A	109.4
O4—Cd1—N1^i^	70.5 (2)	C2—C1—H1A	109.4
N1—Cd1—N1^i^	143.5 (3)	N1—C1—H1B	109.4
C9—N1—C1	114.3 (6)	C2—C1—H1B	109.4
C9—N1—Cd1	109.8 (5)	H1A—C1—H1B	108.0
C1—N1—Cd1	115.7 (5)	C5—C6—C7	122.1 (9)
C9—N1—H1	110.7	C5—C6—H6	118.9
C1—N1—H1	103.5	C7—C6—H6	118.9
Cd1—N1—H1	102.0	C4—C5—C6	118.8 (9)
N1—C9—C10	114.1 (8)	C4—C5—H5	120.6
N1—C9—C8	110.2 (6)	C6—C5—H5	120.6
C10—C9—C8	112.0 (7)	N2—C17—C18	118.3 (13)
N1—C9—H9	106.7	N2—C17—H17	120.8
C10—C9—H9	106.7	C18—C17—H17	120.8
C8—C9—H9	106.7	N2—C21—C20	122.7 (10)
C13—C14—C15	119.1 (13)	N2—C21—C21^i^	118.1 (5)
C13—C14—O3	122.0 (9)	C20—C21—C21^i^	119.2 (8)
C15—C14—O3	118.9 (11)	C19—C20—C21	116.2 (12)
C16—C11—C12	115.8 (11)	C19—C20—C22	126.8 (12)
C16—C11—C10	120.8 (9)	C21—C20—C22	116.7 (14)
C12—C11—C10	123.2 (11)	C18—C19—C20	119.0 (12)
C13—C12—C11	118.4 (11)	C18—C19—H19	120.5
C13—C12—H12	120.8	C20—C19—H19	120.5
C11—C12—H12	120.8	C19—C18—C17	124.7 (14)
C16—C15—C14	121.6 (13)	C19—C18—H18	117.6
C16—C15—H15	119.2	C17—C18—H18	117.6
C14—C15—H15	119.2	C3—O1—H1C	109.5
C15—C16—C11	122.8 (10)	C14—O3—H3	109.5
C15—C16—H16	118.6	C21—N2—C17	118.9 (8)
C11—C16—H16	118.6	C21—N2—Cd1	116.0 (6)
C11—C10—C9	114.9 (7)	C17—N2—Cd1	125.1 (7)
C11—C10—H10A	108.5	O4^i^—C8—O2	125.7 (12)
C9—C10—H10A	108.5	O4^i^—C8—C9	118.5 (7)
C11—C10—H10B	108.5	O2—C8—C9	115.8 (11)
C9—C10—H10B	108.5	C8^i^—O4—Cd1	115.0 (6)
H10A—C10—H10B	107.5	C22^i^—C22—C20	123.5 (8)
C14—C13—C12	121.7 (10)	C22^i^—C22—H22	118.3
C14—C13—H13	119.2	C20—C22—H22	118.3
			
C1—N1—C9—C10	68.7 (9)	Cd1—N1—C1—C2	55.7 (8)
Cd1—N1—C9—C10	−159.4 (6)	C3—C2—C1—N1	69.5 (11)
C1—N1—C9—C8	−164.2 (7)	C7—C2—C1—N1	−109.1 (8)
Cd1—N1—C9—C8	−32.3 (8)	C2—C7—C6—C5	−0.4 (13)
C16—C11—C12—C13	−5.1 (15)	C3—C4—C5—C6	−2.2 (15)
C10—C11—C12—C13	−180.0 (10)	C7—C6—C5—C4	2.1 (15)
C13—C14—C15—C16	6.5 (18)	N2—C21—C20—C19	−3.0 (13)
O3—C14—C15—C16	−173.7 (11)	C21^i^—C21—C20—C19	179.1 (10)
C14—C15—C16—C11	−7.6 (18)	N2—C21—C20—C22	−177.2 (10)
C12—C11—C16—C15	6.7 (16)	C21^i^—C21—C20—C22	4.9 (15)
C10—C11—C16—C15	−178.2 (10)	C21—C20—C19—C18	3.0 (17)
C16—C11—C10—C9	−116.7 (11)	C22—C20—C19—C18	176.5 (14)
C12—C11—C10—C9	57.9 (12)	C20—C19—C18—C17	−1 (2)
N1—C9—C10—C11	54.2 (11)	N2—C17—C18—C19	−1.6 (19)
C8—C9—C10—C11	−71.9 (11)	C20—C21—N2—C17	0.7 (12)
C15—C14—C13—C12	−5.1 (18)	C21^i^—C21—N2—C17	178.6 (9)
O3—C14—C13—C12	175.2 (11)	C20—C21—N2—Cd1	−178.6 (6)
C11—C12—C13—C14	4.7 (18)	C21^i^—C21—N2—Cd1	−0.7 (11)
O1—C3—C2—C7	178.1 (8)	C18—C17—N2—C21	1.6 (13)
C4—C3—C2—C7	0.9 (13)	C18—C17—N2—Cd1	−179.2 (7)
O1—C3—C2—C1	−0.6 (12)	N1—C9—C8—O4^i^	5.4 (13)
C4—C3—C2—C1	−177.8 (8)	C10—C9—C8—O4^i^	133.6 (10)
C2—C3—C4—C5	0.7 (15)	N1—C9—C8—O2	−172.5 (10)
O1—C3—C4—C5	−176.3 (9)	C10—C9—C8—O2	−44.3 (13)
C3—C2—C7—C6	−1.1 (11)	C19—C20—C22—C22^i^	177 (2)
C1—C2—C7—C6	177.6 (7)	C21—C20—C22—C22^i^	−10 (3)
C9—N1—C1—C2	−175.3 (8)		

Symmetry code: (i) *y*, *x*, −*z*.

### Hydrogen-bond geometry (Å, º)

**Table:**

*D*—H···*A*	*D*—H	H···*A*	*D*···*A*	*D*—H···*A*
N1—H1···O1	1.02	2.27	2.992 (10)	126
O6—H6*A*···O2^ii^	0.83	2.51	3.276 (18)	152
O6—H6*B*···O3	0.83	2.43	2.851 (16)	112
O5—H5*B*···O2	0.82	2.00	2.675 (15)	140
O5—H5*A*···O6^iii^	0.83	2.31	2.709 (17)	110
O1—H1*C*···O5^iv^	0.82	1.98	2.652 (11)	138
O3—H3···O4^v^	0.82	1.86	2.640 (9)	159

Symmetry codes: (ii) *x*, *y*+1, *z*; (iii) *y*−1, *x*, −*z*; (iv) *y*, *x*+1, −*z*; (v) −*y*+3/2, *x*+1/2, *z*−1/4.

### Fractional atomic coordinates and isotropic or equivalent isotropic displacement parameters (Å^2^)

**Table:**

	*x*	*y*	*z*	*U_iso_*/U_eq_*
H5B	0.53610	0.83341	-0.03312	-1.5000*
H5A	0.49262	0.79587	0.00596	-1.5000*
H6A	0.71075	1.67885	-0.06649	-1.5000*
H6B	0.65502	1.59306	-0.05379	-1.5000*
H1	0.81784	1.12910	0.00361	-1.5000*
H9	0.71866	0.93508	0.03227	-1.2000*
H12	0.66741	1.08151	-0.08882	-1.2000*
H15	0.57254	1.39986	-0.01802	-1.2000*
H16	0.58588	1.25538	0.02554	-1.2000*
H10A	0.56390	1.00031	-0.00745	-1.2000*
H10B	0.59548	1.07099	0.03600	-1.2000*
H13	0.65867	1.23926	-0.13123	-1.2000*
H4	0.98405	1.41589	0.05806	-1.2000*
H7	0.98855	1.07766	0.12667	-1.2000*
H1A	0.81539	1.03422	0.09265	-1.2000*
H1B	0.74257	1.13120	0.07710	-1.2000*
H6	1.12660	1.20083	0.13947	-1.2000*
H5	1.12698	1.36721	0.10339	-1.2000*
H17	1.16997	0.90939	0.07743	-1.2000*
H19	1.39945	1.12931	0.08438	-1.2000*
H18	1.33013	0.97996	0.10761	-1.2000*
H1C	0.79076	1.35434	0.04265	-1.5000*
H3	0.64690	1.42335	-0.12985	-1.5000*
H22	1.37514	1.30259	0.03087	-1.2000*

### Atomic displacement parameters (Å^2^)

**Table:**

	U^11^	U^22^	U^33^	U^12^	U^13^	U^23^
O5	0.30632	-0.03555	-0.05629	0.08236		
O6	0.30632	-0.03555	-0.05629	0.08236		
CD1	0.05496	-0.01045	-0.00387	0.00387		
N1	0.07071	-0.00960	0.00097	0.00839		
C9	0.08445	-0.02413	-0.00801	0.01661		
C14	0.06406	0.04507	-0.01530	-0.00572		
C11	0.07539	-0.00074	-0.00648	-0.00034		
C12	0.07360	0.02462	-0.01214	-0.00233		
C15	0.06494	0.03013	-0.01113	0.00131		
C16	0.06149	0.02958	-0.01814	-0.01600		
C10	0.08250	-0.01520	-0.00316	0.01360		
C13	0.06021	0.03253	-0.01627	-0.00392		
C3	0.07753	-0.00482	-0.00042	0.00249		
C2	0.04790	-0.00124	0.00797	-0.01036		
C4	0.08564	-0.02337	-0.00646	0.00369		
C7	0.05575	0.00790	0.00220	-0.01002		
C1	0.07208	-0.00825	0.00664	0.00936		
C6	0.07557	0.01449	-0.01915	-0.03346		
C5	0.09689	-0.01618	0.00551	-0.01544		
C17	0.08184	0.03305	-0.02514	-0.02312		
C21	0.07925	-0.02432	0.01955	-0.03695		
C20	0.13837	-0.03174	0.03299	-0.07310		
C19	0.11908	-0.00259	-0.00503	-0.05252		
C18	0.10714	0.02142	-0.02969	-0.03764		
O1	0.12646	-0.01375	-0.02750	0.01621		
O3	0.08593	0.04081	-0.01337	-0.00424		
N2	0.06405	-0.00791	-0.00648	-0.01878		
C8	0.09793	-0.03568	-0.02867	-0.00747		
O2	0.30632	-0.03555	-0.05629	0.08236		
O4	0.07544	-0.01283	0.02229	0.01785		
C22	0.19293	-0.06739	0.03601	-0.06759		
