# Supplementary figures and images for: Structural characterization and DFT study of bis­{(S)-2-[(2-hy­droxy­benz­yl)amino]-3-(4-hy­droxy­phen­yl)propano­ato-κ2 N,O}(1,10-phenanthroline-κ2 N,N′)cadmium(II) tetra­hydrate
Source: Acta Crystallogr E Crystallogr Commun. 2018 Aug 24;74(Pt 9):1339–43. doi: 10.1107/S205698901801157X (PMC6127690; doi:10.1107/S205698901801157X)

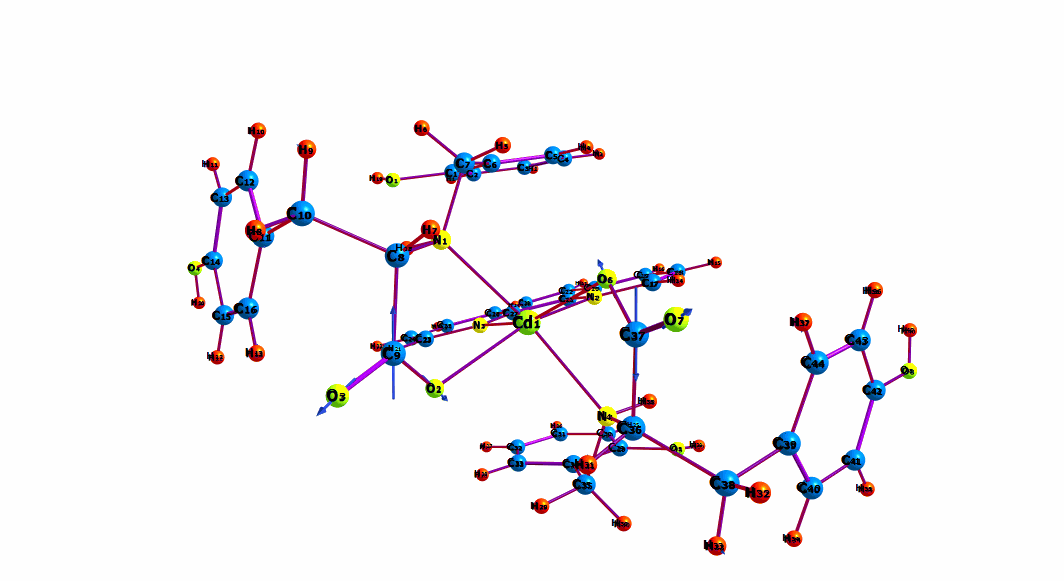

Supplement: Supplementary file 3 [file e-74-01339-sup3.gif]

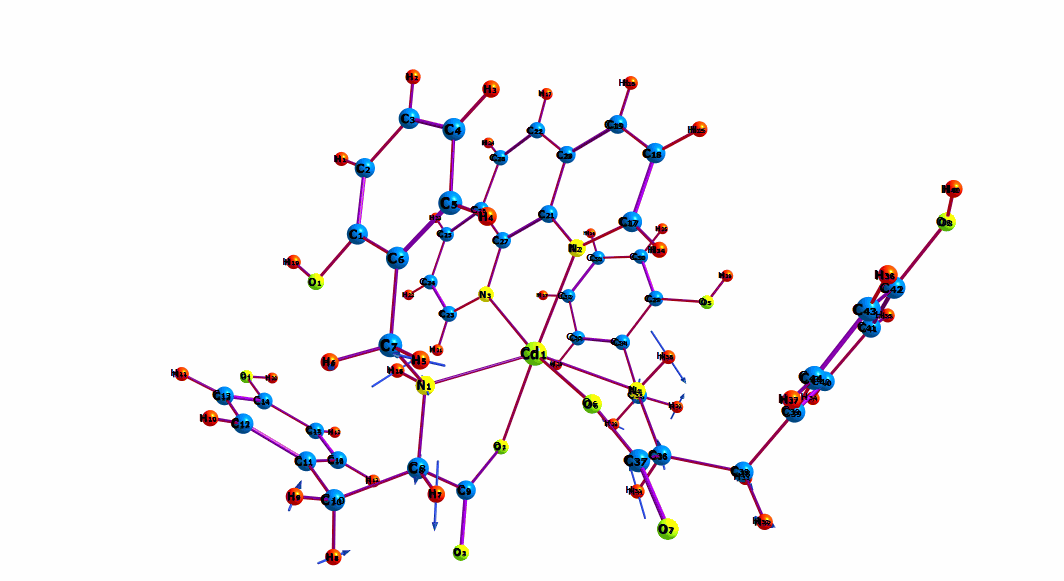

Supplement: Supplementary file 4 [file e-74-01339-sup4.gif]

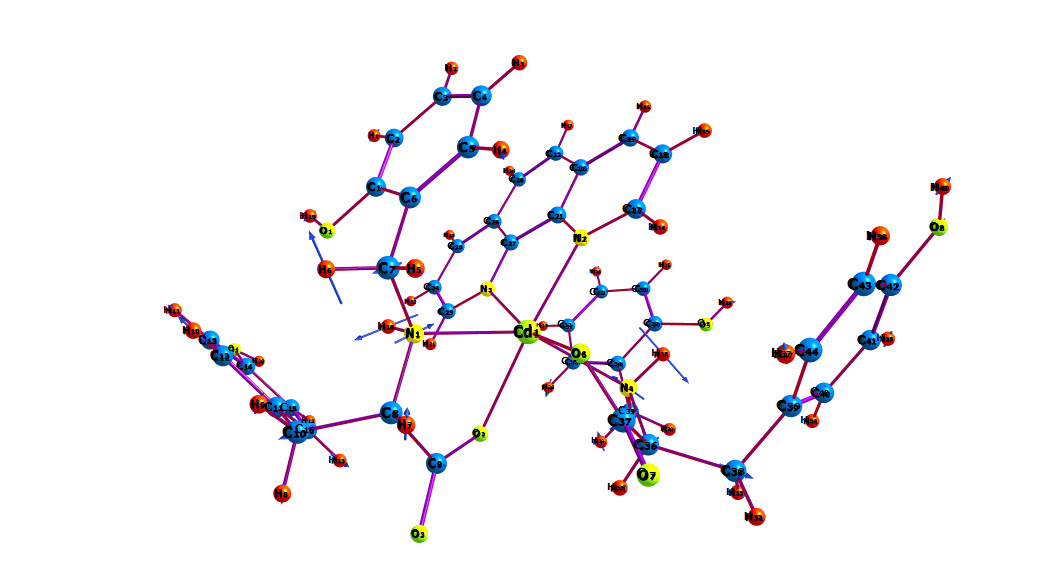

Supplement: Supplementary file 5 [file e-74-01339-sup5.gif]

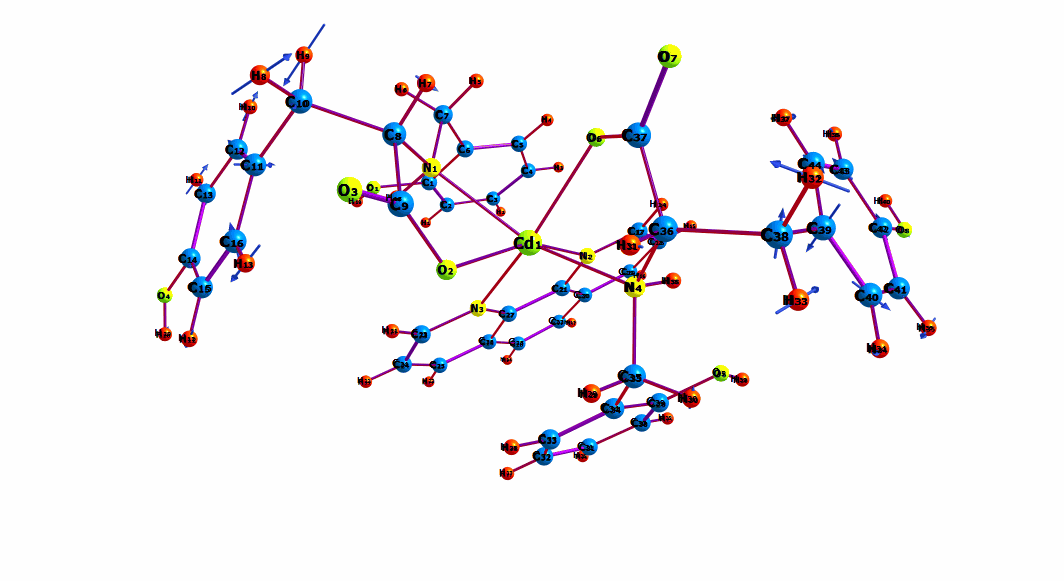

Supplement: Supplementary file 6 [file e-74-01339-sup6.gif]

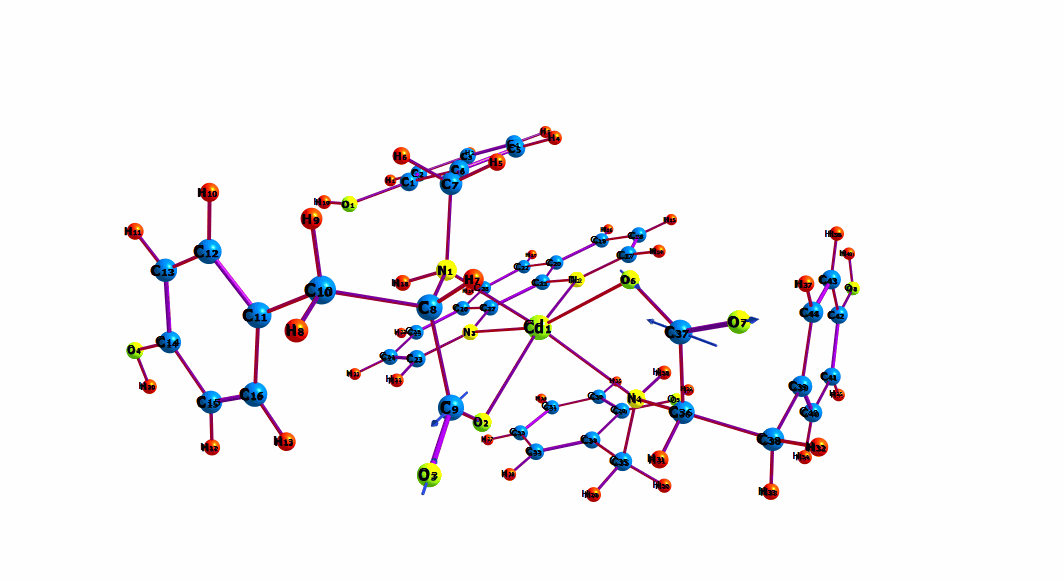

Supplement: Supplementary file 7 [file e-74-01339-sup7.gif]

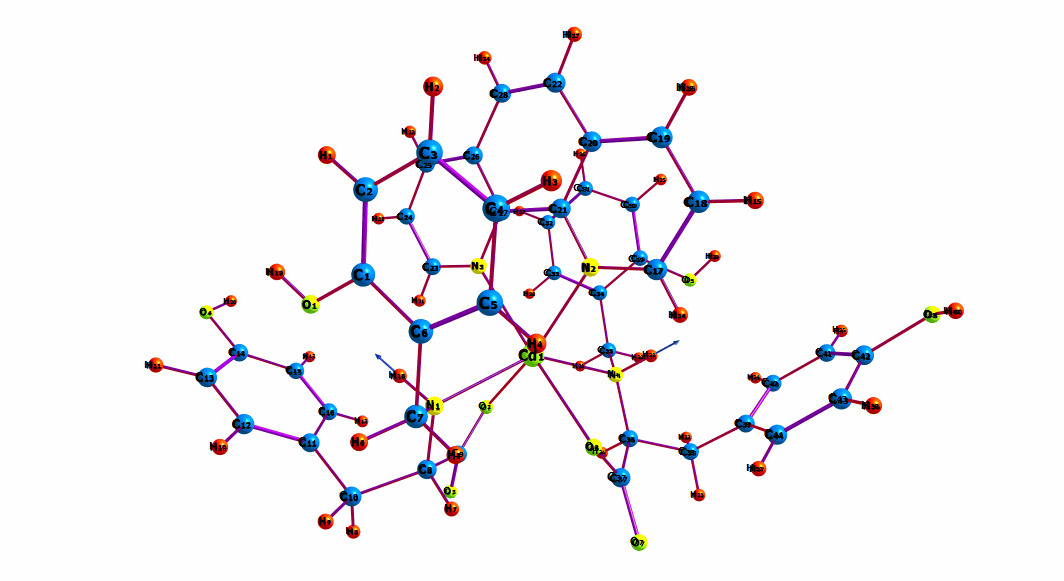

Supplement: Supplementary file 8 [file e-74-01339-sup8.gif]
